# N-Glycoproteomic Profiling Reveals Alteration In Extracellular Matrix Organization In Non-Type Bladder Carcinoma

**DOI:** 10.3390/jcm8091303

**Published:** 2019-08-24

**Authors:** Barnali Deb, Krishna Patel, Gajanan Sathe, Prashant Kumar

**Affiliations:** 1 Institute of Bioinformatics, International Technology Park, Bangalore 560066, India; 2 Manipal Academy of Higher Education (MAHE), Manipal 576104, Karnataka, India; 3 School of Biotechnology, Amrita Vishwa Vidyapeetham, Kollam 690525, India

**Keywords:** urothelial cancer, glycoproteomics, activated pathways, Reactome pathway analysis, molecular subtypes, EMT

## Abstract

Treatment of advanced and metastatic bladder carcinoma is often ineffective and displays variable clinical outcomes. Studying this aggressive molecular subtype of bladder carcinoma will lead to better understanding of the pathogenesis which may lead to the identification of new therapeutic strategies. The non-type bladder subtype is phenotypically mesenchymal and has mesenchymal features with a high metastatic ability. Post-translational addition of oligosaccharide residues is an important modification that influences cellular functions and contributes to disease pathology. Here, we report the comparative analysis of N-linked glycosylation across bladder cancer subtypes. To analyze the glycosite-containing peptides, we carried out LC-MS/MS-based quantitative proteomic and glycoproteomic profiling. We identified 1299 unique N-linked glycopeptides corresponding to 460 proteins. Additionally, we identified 118 unique N-linked glycopeptides corresponding to 84 proteins to be differentially glycosylated only in non-type subtypes as compared to luminal/basal subtypes. Most of the altered glycoproteins were also observed with changes in their global protein expression levels. However, alterations in 55 differentially expressed glycoproteins showed no significant change at the protein abundance level, representing that the glycosylation site occupancy was changed between the non-type subtype and luminal/basal subtypes. Importantly, the extracellular matrix organization pathway was dysregulated in the non-type subtype of bladder carcinoma. N-glycosylation modifications in the extracellular matrix organization proteins may be a contributing factor for the mesenchymal aggressive phenotype in non-type subtype. These aberrant protein glycosylation would provide additional avenues to employ glycan-based therapies and may lead to the identification of novel therapeutic targets.

## 1. Introduction

Bladder carcinoma is typically encountered in older patients [1] and found to be associated with significant mortality, morbidity and cost of treatment [2,3]. Treatment options for aggressive bladder carcinoma have not progressed in the last decades. Nevertheless, increasing high-throughput datasets have increased our knowledge and the understanding of cancer pathogenesis [4]. This led to the identification of the molecular subtypes of bladder carcinoma; although it still lacks the precision as well as in-depth clinical annotation [5]. Post translational modifications (PTMs), such as phosphorylation, modulate a cell’s phenotype and interpose its biological behavior. However, not only phosphorylation but other PTMs, such as glycosylation, are also critical molecular events in cancer pathology. Alterations in the glycosylation of a protein are also associated with malignant transformation of a cell [6]. The typical glycosylation changes are O-linked and N-linked glycosylations. Increased branching of the N-linked glycans has already been reported in certain solid tumors [7,8] involved in cellular and biological processes such as cell–matrix interaction, immune scrutiny, inflammation, cell–cell adhesion, signaling and cellular metabolism [9,10,11,12]. Altered levels of glycosylation are also often observed to be analogous to the overexpression of the major cancer-associated glycoproteins [13]. For example, HER2 (also known as ErbB2 or HER2/neu) is a heavily glycosylated protein that is overexpressed in muscle-invasive bladder carcinoma [14,15]. The overexpression is associated with tumor aggressiveness, poor prognosis and responsiveness to therapy, and could be used as a prognostic marker. However, its glycosylation role in bladder carcinoma is yet to be elucidated [14,16]. Furthermore, N-glycosylated immunoglobulins have also been reported to be a diagnostic biomarker for urothelial carcinomas [17]. Besides, glycan can also modify the functionality of a protein by contributing to its conformational change.

Epithelial-mesenchymal transition (EMT) is also characterized by major changes in the glycosylation pattern of the extracellular matrix (ECM) components as well as cell-surface glycoconjugates [18]. Ding et al. have previously shown that the process of EMT may be used to explain the impact of atypical glycans expressed by invasive epithelial cells [19]. Glycosylation of ECM proteins are reported in breast [20], lung [21], ovarian [22], neuroblastoma [23] and prostate cancer [24] which have been associated with invasion, migration and cancer progression. Aberrant glycosylation has also been reported in bladder cancer [25]. Compromised N-Acetylglucosaminyltransferases (GnTs) expression has been shown to amend the N-glycans branching resulting in bladder cancer prognosis [26]. Specifically, increased GnT-III, N-glycans bisection and GnT-IV expression were associated with higher disease stage and grade in bladder cancer patients [27]. However, it would be remarkable to correlate levels of N-linked glycosylation in bladder carcinoma molecular subtypes. This would immensely impact on the clinical outcome of the aggressive subtype of bladder carcinoma. A study by Warrick et al. categorized three molecular subtypes of bladder carcinoma cell lines: Luminal (RT112, SW780), basal (VMCUB-1) and non-type (J82, T24 and UMUC3) [28]. We have recently shown that the non-type molecular subtype of bladder carcinoma cells exhibits the most aggressive phenotype and are more “mesenchymal-like” [29]. We further extended our study to address the post-translational addition of oligosaccharide residues in all three subtypes of bladder carcinoma and its correlation to disease pathology.

In this study, we conducted a comprehensive quantitative proteomic and glycoproteomic analysis of the different molecular subtypes of bladder carcinoma cell lines (RT112, SW780, VMCUB-1, J82, T24 and UMUC3). We employed a liquid chromatography tandem-mass spectrometry (LC-MS/MS) based approach to investigate protein expressions and their glycosylation levels. To our knowledge, this is the first report on the profiling of the glycoproteins in bladder carcinoma and further dissection of it to check the glycosylation pattern across different subtypes.

## 2. Experimental Section

### 2.1. Cell Culture

Bladder carcinoma cell lines, SW780, RT112, VMCUB-1, T24, J82, and UMUC3 were cultured in Dulbecco’s Modified Eagle’s Medium (DMEM; with high glucose, HIMEDIA), supplemented with 10% fetal bovine serum (FBS) and 1% penicillin/streptomycin. Cells were maintained in a humidified incubator at 37 °C and 5% CO_2_.

### 2.2. Cell Lysis and Protein Extraction

Cell lines were grown to 70% confluence, starved in serum-free medium for 12 h, and then lysed in cell lysis buffer (2% SDS in 50 mM triethyl ammonium bicarbonate (TEABC)) with sonication. Protein concentration was estimated using the BCA method (Pierce; Waltham, MA, USA). 300 µg of protein from each cell line were reduced and alkylated using 5 mM dithiothreitol (DTT) and 20 mM iodoacetamide (IAA), respectively. The proteins were precipitated using ice cold acetone. The precipitated proteins were dissolved in the 50 mM TEABC and used for further in-solution digestion.

### 2.3. In-Solution Trypsin Digestion of Proteins and TMT Labeling

Protein digestion was carried out using lys-C (1:100) for 4 hrs, followed by trypsin (1:20) at 37 °C for 12–16 h. Resulted peptides were enriched using Sep-Pak C_18_ material. Peptides from the respective bladder carcinoma samples were labeled using 6-plex Tandem Mass Tag (TMT) as per the manufacturer’s instruction (Catalog # 90110, Thermo Fisher Scientific, Rockford, IL, USA). Peptides derived from T24, J82, RT112, VMCUB-1, UMUC3 and SW780, and were labeled with 126, 127, 128, 129, 130 and 131 respectively. After labeling, the reactions were quenched and labels pooled together.

### 2.4. N-Glycan Enrichment

Glycopeptides from the tryptic peptide digest were enriched using solid-phase extraction of glycosite-containing peptides (SPEG) [30]. In this method carbohydrates in the glycopeptides were oxidized into the aldehydes, which then formed covalent hydrazone bonds with hydrazide groups immobilized on a solid support. Non-glycosylated peptides were washed from the resins and glycosylated peptides remain on the solid support. These attached N-linked glycopeptides were released from the resins with the treatment of the PNGase F. This PNGase F action led to the conversion of the glycosylated asparagine to aspartic acids, generating a 1-unit mass shift at the site of glycosylation, which was detectable using a high accuracy mass spectrometer and is diagnostic for the glycosylation site. Here, we used a combination of the isobaric TMT labeling method for quantitation along with the SPEG method for the identification of the N-linked glycosylated proteins, the site(s) of N-linked glycosylation, and the relative quantity of the identified glycopeptides in single analysis.

90% of the TMT-labeled tryptic peptides (1 mg) were dissolved in 5% ACN in 0.1% TFA followed by adding 1/10 of the final volume of 100 mm sodium periodate to the samples. These peptides were incubated in the dark at room temperature for 1 hour with gentle shaking. After oxidation, peptides were cleaned using C_18_ clean-up. For the activation of beads, 200 µL of the 50% hydrazide resin (Bio-Rad Laboratories, Hercules, CA, USA) were used. These beads were further washed three times with 1 ml deionized water. Following this, peptides along with 1% Aniline were added to the beads (Ph < 6). The peptides were incubated with beads for 1 h at room temperature with gentle shaking. To avoid non-specific binding, beads were washed with 50% acetonitrile followed by 1.5 M NaCl and with deionized water three times. Finally, the resins were resuspended in 200 µL of 25 mM NH4HCO3 buffer incubated with PNGaseF (New England Biolabs, Ipswich, MA, USA) at 37 °C overnight with gentle shaking. The supernatant containing N-linked glycopeptides were collected and dried using vacufuge. These enriched N-linked glycopeptides were directly used for the mass spectrometric analysis.

### 2.5. LC-MS/MS Analysis

The peptides and enriched glycopeptides were analyzed on Q Exactive HF-X Hybrid Quadrupole-Orbitrap mass spectrometer (Thermo Scientific, Bremen, Germany) interfaced with Dionex Ultimate 3000 nanoflow liquid chromatography system. Peptides were separated on an analytical column (75 µm × 50 cm, RSLC C_18_) at a flow rate of 300 nl/min using a gradient of 8%–35% solvent B (0.1% formic acid in 90% acetonitrile) for 105 min. The total run time was set to 120 min. The mass spectrometer was operated in a data-dependent acquisition mode. The precursor scan MS scan (from m/z 350–1600) was acquired in the Orbitrap at a resolution of 120,000 at 200 m/z. The automatic gain control (AGC) target for MS1 was set as 3 × 10^6^ and ion filling time set at 50 ms. The most intense ions with charge state ≥ 2 were isolated and fragmented using HCD fragmentation with 34% normalized collision energy and detected at a mass resolution of 45,000 at 200 m/z. The AGC target for MS/MS was set as 2 × 10^5^ and ion filling time set at 100 ms, while dynamic exclusion was set for 30 s with a 10-ppm mass window.

### 2.6. Data Analysis

The acquired mass spectrometry data were searched against Human RefSeq protein database (version 81, containing protein entries with common contaminants) using SEQUEST search algorithm through Proteome Discoverer platform (version 2.1, Thermo Fisher Scientific, San Jose, CA, USA). The following search parameters were used: Two missed cleavages allowed, trypsin as cleavage enzyme, a tolerance of 10 ppm on precursors and 0.02 Daltons on the fragment ions. Fixed modification includes: Carbamidomethylation at cysteine, TMT 6-plex (+229.163) modification at N-terminus of peptide and lysine and variable modification as oxidation of methionine and deamination of asparagine and glutamine. Data were also searched against a decoy database and filtered with a 1% false discovery rate (FDR). Posterior error probability was calculated for individual peptide spectral match (PSM) using percolator, providing statistical confidence for each spectral match. Glycopeptides were further filtered for consensus sequence of N-linked glycosylation motif NXS/T. Protein centric glycosylation sites were inferred using *in house* PERL script. The relative abundance of N-glycoproteins was calculated.

### 2.7. Statistical Analysis

For the identification of glycopeptides differentially glycosylated between non-type and luminal/basal subtypes, two sample “*t*-test” with unequal variances (*p* < 0.05) was used. This led to the identification of a list of glycoproteins which were further used for clustering, pathway and network analysis.

### 2.8. Clustering the Molecular Subtypes

All the replicates in the glycoproteomics dataset (four replicates) were considered for identifying the differentially glycosylated peptides in the non-type subtype. A non-parametric *t*-test was conducted on the normalized abundance values to determine statistical significance of the differentially glycosylated peptides in the non-type subtype as compared with the luminal/basal subtype. Differentially glycosylated peptides (*p* ≤ 0.05) were further considered for supervised clustering using Morpheus (Broad Institute, Cambridge, MA, USA) (https://software.broadinstitute.org/morpheus).

### 2.9. Protein-Protein Interaction Network Analysis

Interaction network was analyzed using STRING functional protein association network (https://string-db.org; version: 11.0; University of Zurich, Zurich, Switzerland) [31]. The input was the set of differentially glycosylated proteins (*p* ≤ 0.05) in the non-type subtype as compared to the luminal/basal and was set to highest confidence (0.90) of active interaction. The disconnected nodes were hidden, and K-means clustering was conducted to identify three clusters in the dataset.

### 2.10. Reactome Pathway Analysis

Reactome analysis tool (http://reactome.org) was used to identify the enriched pathways in the non-type using the set of differentially glycosylated peptides (*p* ≤ 0.05) (https://reactome.org/PathwayBrowser/#TOOL=AT). The “extracellular matrix organization pathway” was enriched and a schematic pathway map was annotated using Adobe Illustrator (vCS5.1; AdobeSystems, SanJose, CA, USA) for illustration.

## 3. Results

### 3.1. Quantitative Analysis of N-Glycoprotein in Bladder Carcinoma Cell Lines

**Figure 1:**
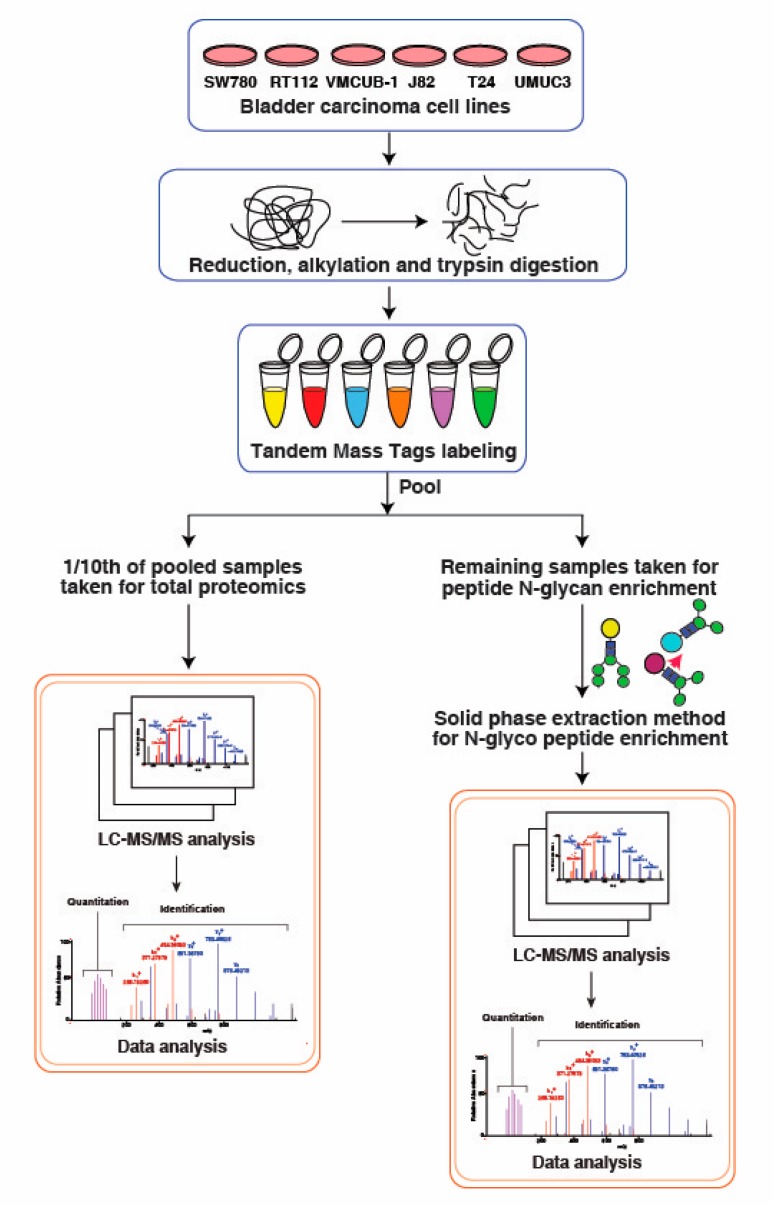
Workflow for the quantitative global proteomics and glycoproteomics analysis of bladder carcinoma cell lines. For sample processing, proteins were extracted from the bladder carcinoma cell lines and digested using trypsin. Each cell line was tagged using the 6-plex Tandem Mass Tag (TMT) labeling kit and lyophilized. One tenth of the samples were taken for global proteomics and the remainder was enriched using the glycopeptide enrichment protocol. The samples were run on Q Exactive HF-X Hybrid Quadrupole-Orbitrap mass spectrometer and MS2-based quantitation was achieved. The files were searched against Mascot and Sequest HT search engines. Data were acquired in technical replicates.

**Figure 2:**
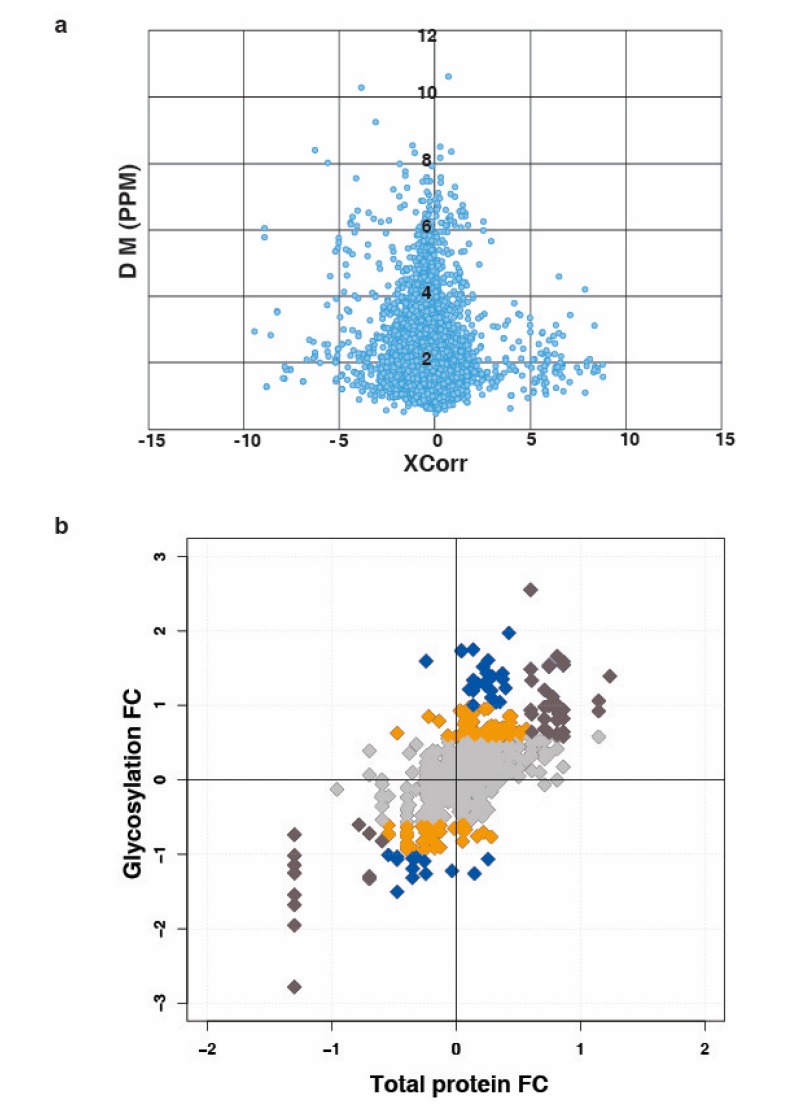
(**a**) Distribution of the mass error from the parent ion measurement against the SEQUEST Xscore; (**b**) global presentation of proteomic and respective glycosylation occupancy. Yellow datapoints represent differential glycosylation occupancy with fold change ≥ 1.5 and blue datapoints with fold change ≥ 2 on proteins that are unchanged in proteomic data. Brown datapoints represent proteins and glycosylation occupancy with similar dysregulation pattern (overexpressed/increased glycosylation occupancy and/or downregulated/reduced glycosylation occupancy). Grey datapoints represent proteins that are unchanged in both datasets.

To correlate protein level and changes at glycosylation occupancy, we performed N-glycosylation profiling across bladder cancer cell lines. SW780, RT112, VMCUB-1, J82, T24 and UMUC3 cells were used in three different categories, categorized as luminal, basal and non-type based on the previous publication [28]. A TMT-based quantitative approach was employed along with N-linked glycopeptides enrichment using hydrazide chemistry. To eliminate the variation, we used 10% TMT labeled peptides for proteomic analysis and the remaining 90% were further utilized for the glycopeptides enrichment using solid phase extraction of glycopeptides (SPEG). The workflow for the experiment is outlined in Figure 1. LC-MS/MS data were processed and searched against databases using SEQUEST algorithm. The identified deamidated peptides with NXS/T motifs, where X is any amino acid except proline, were only used for further analysis. Other modified sites were considered as experimental artifact and discarded. We identified 1299 unique N-glycopeptides from the bladder cancer cell lines. The identified N-glycopeptides and their total protein expression along with the quantitation are presented in the Supplementary Table S1). After database searches, we have plotted the distribution of the mass error from the parent ion measurement against the SEQUEST Xscore. Majority of the data falls within five part per million mass error. The data is depicted in Figure 2a.

### 3.2. N-Linked Glycosylation in Aggressive Non-Type Bladder Carcinoma

From the identified 1299 glycopeptides, 339 N-linked glycosylated peptides were identified as dysregulated (1.5-fold change) in the non-type as compared to the luminal/basal (Supplementary Figure S1). Although we identified the changes in 339 glycopeptides, it is not clear whether the changes were because of glycosylation site occupancy or changes in the abundance in the protein levels. To address this, we compared the fold change from non-type to the luminal/basal for the N-glycosylation fold change against the protein fold changes using quadrant plot (Figure 2b). Interestingly, we identified 55 proteins which were differentially glycosylated without significant changes in global protein levels (represented with blue dots in Figure 2b) (Supplementary Table S2).

### 3.3. Unique N-Linked Glycosylation Signature in Aggressive Non-Type Bladder Carcinoma Cell Lines

**Figure 3:**
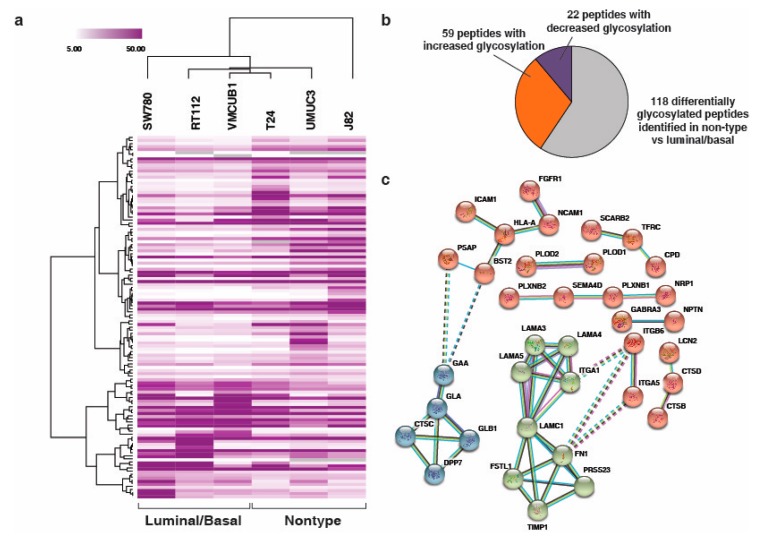
(**a**) Supervised clustering of the molecular subtypes of bladder carcinoma cell lines. A *t*-test was conducted on the glycoproteomic data that identified 118 N-linked glycopeptides (corresponding to 84 proteins) which were differentially glycosylated in the non-type cell lines (T24, J82, and UMUC3) as compared with the luminal/basal subtype (SW780, RT112, and VMCUB-1) (*p* ≤ 0.05); (**b**) pie chart depicting the number of N-linked glycopeptides which depicts increased and decreased glycosylation in the non-type cell lines (*p* < 0.05); (**c**) protein–protein interaction network enriched in the non-type cells with highest confidence (0.90) acquired using STRING functional protein association network tool.

In our previous study, we have shown the phosphoproteomic profiling of bladder subtype and identified a unique signature for the aggressive non-type bladder carcinoma cells [29]. In this study, we further sought to evaluate the glycosylation specific signatures for the aggressive non-type cells. We performed a non-parametric *t*-test, to check the differential N-linked glycosylation levels between the luminal/basal and the non-type subtype. We identified a total of 118 N-linked glycopeptides to be differentially glycosylated (*p* < 0.05) corresponding to 84 glycoproteins (Supplementary Table S3). Supervised clustering of these differentially N-linked glycosylated proteins reveals distinct glycosylation signature (Figure 3a). Of the 118 signature glycopeptides, a total of 59 glycopeptides were observed to have increased and 22 had decreased N-linked glycosylation (> or <1.5 fold) in the non-type as compared to the luminal/basal (Figure 3b).

### 3.4. Interaction Network Clusteres in the Non-Type Bladder Carcinoma Cell Lines

The 84 glycoproteins that were differentially glycosylated (*p* < 0.05; between non-type and luminal/basal) were considered for the network analysis. Network analysis revealed three major clusters of 36 interacting proteins (Figure 3c). These clusters clearly depicted key hub proteins such as Laminin Subunit Gamma 1 (LAMC1), Alpha-galactosidase A (GLA) and HLA class I histocompatibility antigen, A-2 alpha chain (HLA-A). LAMC1 interacted with Laminin subunit alpha-5 (LAMA5) and Integrin alpha-1 (ITGA1) which were interconnected with Laminin subunit alpha-5 (LAMA3) and Laminin subunit alpha-5 (LAMA4). Also, LAMC1 interacts with Follistatin-related protein 1 (FSTL1) and Fibronectin (FN1) which interconnect with Serine protease 23 (PRSS23) and Metalloproteinase inhibitor 1 (TIMP1). FN1 also interacts with Integrin beta-6 (ITGB6) and Integrin alpha-5 (ITGA5). GLA interacts with Lysosomal alpha-glucosidase (GAA), Beta-galactosidase (GLB1) and Dipeptidyl peptidase 1 (CTSC) which also interconnect with Dipeptidyl peptidase 2 (DPP7). Thirty-two proteins were identified to be an integral part of the membrane or/and cell surface protein or/and a part of the extracellular region (Supplementary Figure S2).

### 3.5. Reactome Pathway Analysis Identified Dysregulation of Extracellular Matrix Organization Pathway Enriched in the Non-Type Bladder Carcinoma

**Figure 4:**
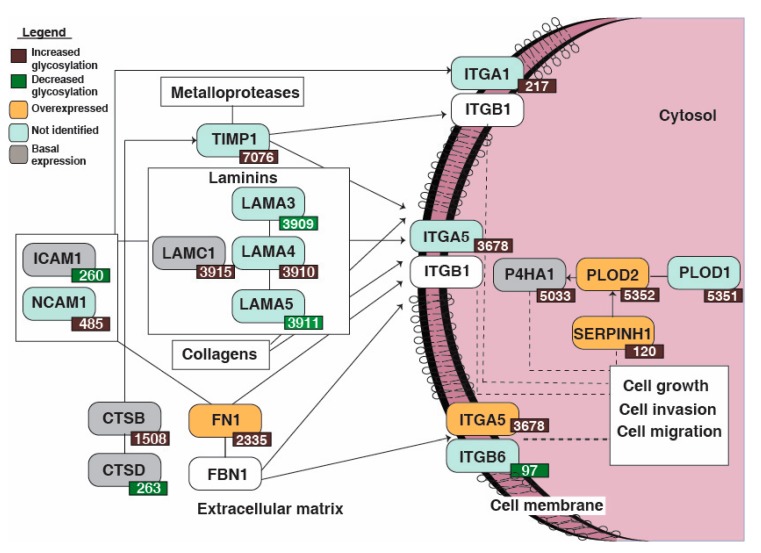
Schematic diagram of enriched extracellular matrix organization pathway in non-type subtype of bladder carcinoma cell lines. Reactome pathway analysis lead to the identification of extracellular matrix organization signaling to be enriched in the aggressive molecular subtype. The dysregulated glycoproteins and corresponding identified global protein expressions are highlighted in the pathway.

Reactome pathway analysis was conducted with the 118 differentially glycosylated peptides corresponding to 84 proteins which led to the identification of extracellular matrix organization pathway (*p* = 9.88E-9, FDR = 2.96E-7). A schematic representation of the pathway is as depicted in Figure 4. Seventeen proteins were enriched in the pathway which were significantly dysregulated in the non-type subtype (Table 1).

The glycosylation levels of ITGA5, Procollagen-lysine, 2-oxoglutarate 5-dioxygenase 2 (PLOD2), Serpin H1 (SERPINH1), Prolyl 4-hydroxylase subunit alpha-1 (P4HA1), FN1, CTSB, ITGA5 and LAMC1 were increased (1.5-fold) and were upregulated in the non-type cells. Neural cell adhesion molecule 1 (NCAM1) and LAMA4 showed only increased glycosylation (1.5-fold) and were not identified in the global proteomics data. (CTSD) and (ICAM1) showed decreased glycosylation and were downregulated (1.5-fold). ITGB6 also showed decreased glycosylation but was not identified in the total proteomics data.

## 4. Discussion

Understanding the molecular subtypes of bladder carcinoma is of pronounced significance as it can help in gaining knowledge on the disease pathogenesis and determining personalized therapy. Molecular subtypes have mostly been defined and studied by analyzing gene expression data based on the enrichment of specific genetic alterations and gene expression profiles. However, a comprehensive and quantitative glycoproteomics study on bladder carcinoma has still not been reported. In this study, we have provided the most comprehensive quantitative proteomic and glycoproteomic representation of bladder carcinoma cell lines to-date. Our study identified 1299 N-linked glycopeptides in the bladder cancer cell lines. We also combined bioinformatics approaches with glycoproteomic analysis of bladder carcinoma cell lines to identify the highly enriched cellular signaling pathways.

For better understanding, we have integrated N-glycoproteome and global proteomics data for the cell lines and compared the abundance values between non-type and luminal/basal cells. The distribution of this data showed that most of the specific glycosite changes were complemented by change in protein abundance. However, 55 glycoproteins were observed to go through alterations in glycosylation without accompanying changes in the protein level between the non-type as compared to the luminal/basal cells. Key proteins among them being ITGB1, LAMC1, P4HA1, CTSB and Laminin subunit beta-2 (LAMB2). ECM components such as collagens, laminins, and fibronectins are the major ligands that bind and activate integrin receptors. It also helps in mediating cell–cell interactions by binding additional cell receptors or other soluble molecules. Twenty impending glycosylation sites (N-linked glycosylation) have been reported which are essential for integrin heterodimerization, stabilization of conformation, expression at the cell membrane, and interaction with ligands [32,33]. ITGB1 has been reported to harbor an increased and branched N-linked glycosylation (6-N-acetylglucosamine (b1,6-GlcNAc)) which is associated with an increase in cell migration as well as invasion (mostly when the branches are sialylated) [34]. N-acetylgalactosaminyltransferase 1 (GALNT1) overexpression has been reported for the aberrant glycosylation of integrin α3β1 which leads to the change in the conformation of integrin and further induces focal adhesion kinase (FAK) activation in bladder cancer cells [35]. This also reveals the concordance of our previous findings where we identified the activation of integrin signaling in the non-type bladder carcinoma. Targeting integrins have shown to decrease angiogenesis, tumor growth and metastasis in other cancers including bladder carcinoma [36,37,38]. FAK activation has also been reported to regulate bladder cancer invasion and migration which also makes it a favorable therapeutic target for bladder carcinoma [39]. LAMC1 is known to be involved in tumor cell proliferation, growth, angiogenesis, invasion and migration [40,41]. Proteins involved in ECM like P4HA2 and PLOD2 mediate remodeling of ECM composition, alignment, and mechanical properties in response to hypoxia [42]. CTSB, a multifunctional proteolytic enzyme, plays an important role in cancer progression. Its increased and redistributed expression pattern in human cancers is considered a marker for invasion and metastasis of various carcinoma cells [43,44]. LAMB2 is yet another important protein which is thought to mediate the attachment, migration and interact with other extracellular matrix proteins [45]. It is known to be a heavily glycosylated protein. In tumorigenic transformation, the cells undergo abnormal levels of glycosylation [46]. Since the ECM is composed of various glycoproteins and polysaccharides, therefore, the mechanism for glycan biosynthesis is also mostly dysregulated. This may contribute to the changes in glycosylation of ECM proteins and can modulate phenotypic characteristics in the cells such as invasiveness. It is also reported to confer multi-drug resistance in a few cancers [46]. Further validative studies are prerequisite to substantiate such supposition.

A variation in the glycosylation levels contributing to EMT has been recently discussed in many reports. One of the most well-established examples is the glycosylation of E-cadherin in gastric cancer [10]. The overexpression of branched-N-glycan structures interferes with epithelial cadherin-mediated cell–cell adhesion and thus promotes tumor cell detachment and invasion. However, our understanding of these composite biological systems remains restricted by the existing analytical technologies.

The differentially glycosylated proteins in non-type compared to the luminal/basal cells were used for the pathway analysis. The Reactome pathway analysis was conducted with the 84 differentially glycosylated proteins that led to the identification of extracellular matrix organization pathway enriched in the non-type subtype. We identified 17 significantly altered proteins that were involved in the ECM pathway. In our earlier study, we have shown that the luminal/basal molecular subtypes revealed epithelial phenotype, whereas aggressive non-type molecular subtype were mesenchymal. It is a well-established fact that cells engaged in EMT undergo metabolic reprogramming which further leads to significant aberration in glycosylation of the extracellular matrix (ECM) components and cell-surface glycoconjugates [18]. Specific N-glycan alterations are coupled with EMT that contributes to cell migration [47]. In our study, we identified ECM proteins to be dysregulated in non-type subtype, which may activate a surfeit of signals eventually regulating EMT during tumorigenesis [48]. The agglomerate efforts of the ECM proteins and changes in their glycosylation could orchestrate invasive mesenchymal-like aggressive characteristics in non-type bladder cells. Our study corroborates previous findings which suggest that the glycosylation changes are often important for cells to undergo EMT. However, it is important to note that the non-type cell population may not include a pure population of only mesenchymal-like cells but may also have a population of hybrid cells (hybrid E/M cells). However, growing data of evidence suggests that not only the mesenchymal cells but also hybrid epithelial/mesenchymal cells have high tumor-initiation capabilities and metastatic potential. The hybrid phenotype has been hypothesized to be even the most aggressive as they deliver a miscellany of paracrine and autocrine signaling, cellular and extracellular attachments and proliferative capacity [49].

A systematic exploration of glycan structures is necessary for the understanding of their roles in the epithelial to mesenchymal transition in bladder carcinoma. The role of site-specific glycosylation needs to be investigated in detail to understand their role in tumorigenesis. More advancement in the high throughput-based LC-MS/MS along with the data analysis pipeline is needed for future analysis. It is now prerequisite to produce glycosite specific antibodies as well as adopt mass spectrometry-based targeted proteomics assays for a deeper validation of differentially glycosylated proteins.

## 5. Conclusions

We present the first quantitative glycoproteomics of bladder carcinoma cells. We provide a profile of the differentially regulated N-linked glycoproteins in the aggressive molecular non-type subtype of bladder carcinoma. Our glycoproteomic analysis identified ECM proteins which may contribute to the aggressive nature of the non-type cells. Future studies on the glycosylation sites as well as the glycan structures could help in additional mechanistic studies.

## Figures and Tables

**Table 1:** N-linked glycosylation levels and their corresponding expression of proteins involved in extracellular matrix organization pathway.

Gene Symbol	Glycosylation Site	Glycosylation Fold Change	Total Protein Fold Change *
LAMC1	3915	2.0	1.1
SERPINH1	120	2.8	1.5
PLOD2	5352	3.0	1.8
TIMP1	7076	1.9	NI
PLOD1	5351	1.8	NI
LAMA3	3909	0.7	NI
P4HA1	5033	2.7	1.3
LAMA4	3910	2.0	NI
CTSB	1508	2.1	1.3
ITGA1	217	1.6	NI
LAMA5	3911	0.7	NI
CTSD	263	0.5	0.7
ITGB6	97	0.3	NI
ITGA5	3678	3.2	1.8
FN1	2335	2.0	2.2
NCAM1	485	2.3	NI
ICAM1	260	0.4	0.7

* NI-not identified.

## Supplementary Materials

The following are available online at https://www.mdpi.com/2077-0383/8/9/1303/s1, Figure S1: Identification of dysregulated N-glycosylation in bladder carcinoma cell lines; Figure S2: Interaction of 32 proteins identified to be a part of integral part of the membrane or/and cell surface protein or/and a part of the extracellular region; Table S1: Quantification of N-glycopeptides and their total protein expression fold change in bladder carcinoma cell lines; Table S2: Differentially N-glycosylated proteins without significant changes in their global protein expression levels; Table S3: Differentially glycosylated (*p* < 0.05) N-linked glycopeptides in the non-type subtype of bladder carcinoma.
