# Supplementary material for: N-Glycoproteomic Profiling Reveals Alteration In Extracellular Matrix Organization In Non-Type Bladder Carcinoma
Source: J Clin Med. 2019 Aug 24;8(9):1303. doi: 10.3390/jcm8091303 (PMC6780497; doi:10.3390/jcm8091303)
Supplement: Supplementary file 1 [file jcm-08-01303-s001.zip › jcm-557887-supplementary/Supplementary figures_JCM_Deb et al., 2019.pdf]

N-Glycosylation profile reveals alteration in extracellular matrix organization pathway in non-type bladder carcinoma

Barnali Deb, Krishna Patel, Gajanan Sathe* and Prashant Kumar*

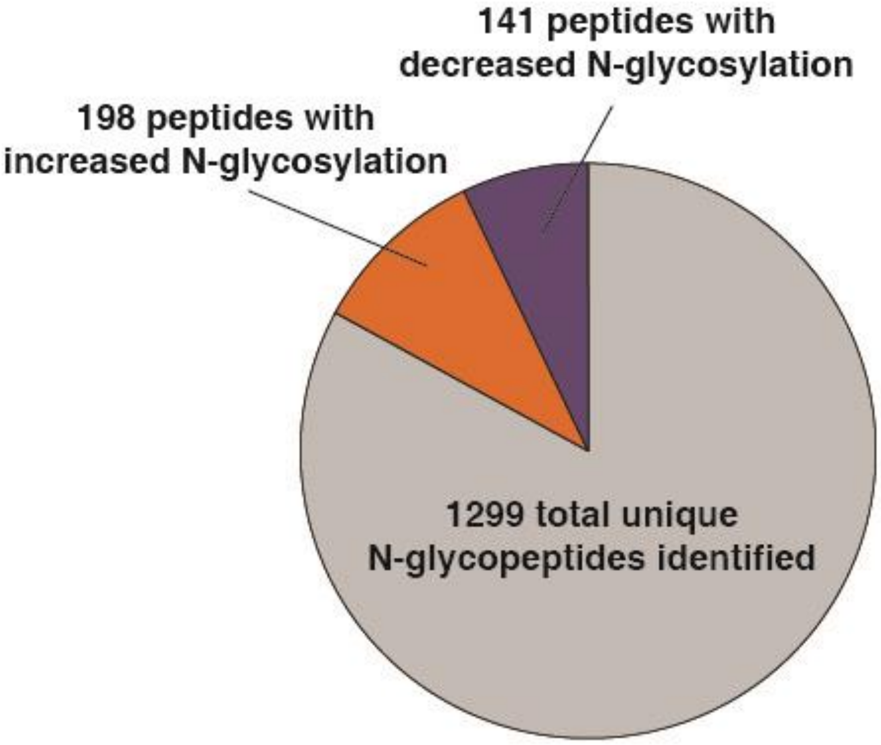

Supplementary figure S1: Identification of dysregulated N-glycosylation in bladder carcinoma cell lines.

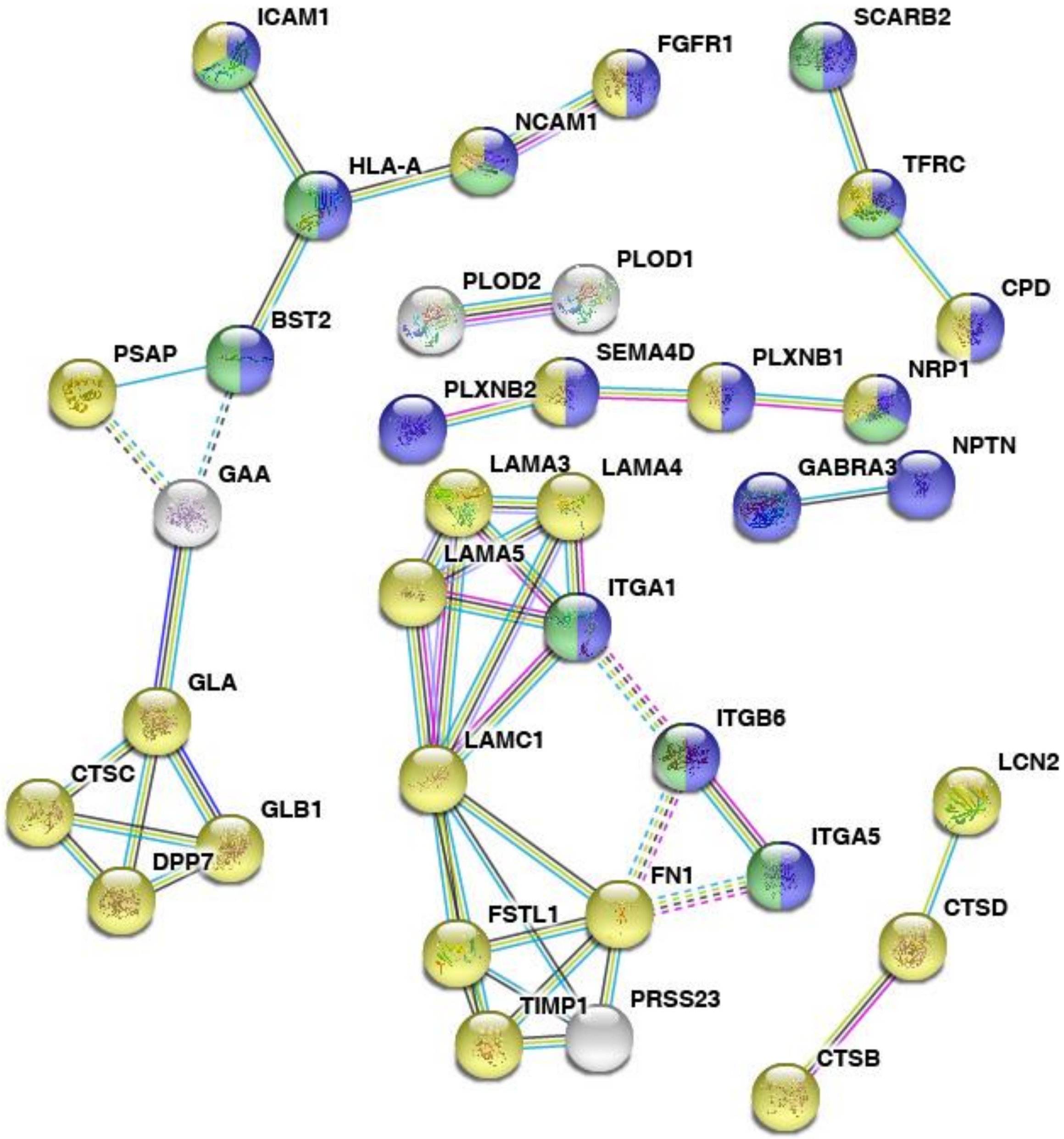

Supplementary figure S2: Interaction of 32 proteins identified to be a part of integral part of the membrane or/and cell surface protein or/and a part of the extracellular region.
